# Most species are not limited by an Amazonian river postulated to be a border between endemism areas

**DOI:** 10.1038/s41598-018-20596-7

**Published:** 2018-02-02

**Authors:** Sergio Santorelli, William E. Magnusson, Claudia P. Deus

**Affiliations:** 1 Programa de Pós graduação em Ciências Biológicas, Instituto Nacional de Pesquisas da Amazônia, Av. André Araújo, 2.936, Petrópolis, CEP 69.067–375 Manaus, Amazonas Brazil; 2 Centro de Estudos Integrados da Biodiversidade Amazônica, Av. André Araújo, 2.936, Petrópolis, CEP 69.067–375 Manaus, Amazonas Brazil; 3 Coordenação de Pesquisas em Biodiversidade, Instituto Nacional de Pesquisas da Amazônia, Av. André Araújo, 2.936, Petrópolis, CEP 69.067–375 Manaus, Amazonas Brazil

## Abstract

At broad scales in the Amazon, it is often hypothesized that species distributions are limited by geographical barriers, such as large rivers (river-barrier hypothesis). This hypothesis has been used to explain the spatial-distribution limits of species and to indicate endemism areas for several phylogenetic lineages. We tested the ability of the river-barrier hypothesis to explain patterns of species diversity and spatial-distribution limits for 1952 easily-detected species in 14 taxonomic groups that occur around the Madeira River, and our results indicate that the hypothesis that the Madeira River is the border between endemism areas and explains much of the diversity found in the region is inappropriate for >99% of species. This indicates that alternative hypotheses should be proposed to explain the limits of distributions of species around the Madeira River, as well as a revision of the criteria that are used to determine species-endemism areas.

## Introduction

Presence or absence of individuals of a species in the Amazon can be attributed to multiple factors. At local scales, habitat characteristics have been identified as the main determinants of the distribution of various of plants^1–4^, lizards^5^, anurans^6,7^, snakes^8^, ants^9^, mammals^10–12^, termites^13^ and birds^14,15^. However, at broader scales, it is often hypothesized that species distributions are mainly related to dispersal limitation caused by geographical barriers, such as large rivers^16,17^. This explanation is commonly referred to as the “river-barrier hypothesis”.

Wallace^18^ was one of the first to hypothesize that the distributions of Amazonian species could be limited by large Amazonian rivers, such as the Negro, Amazon and Madeira Rivers. According to the modern interpretation of this hypothesis, large rivers are expected to subdivide a population to the point of preventing gene flow between individuals in different areas and to promote genetic divergence between them, increasing the opportunity for allopatric speciation^19,20^. If this hypothesis is correct, it is expected that (i) sister species or lineages will be on opposite river banks^21–23^, (ii) the similarity in species composition will be greater in localities on the same bank (adjacent sites) than sites on opposite banks separated by the same distance^24–27^ and (iii) the boundaries of species distributions will coincide with large rivers^21–28^.

The river-barrier hypothesis has been used to explain the spatial-distribution limits of species and to indicate possible endemism areas^29,30^ for several phylogenetic lineages in the several taxa in the Amazon (e.g. primates^23,24^, lizards^17,28^, anurans^16,17,25^, butterflies^21^, birds^22,26,27,31^). The hypothesized endemism areas delimited by rivers have been used as surrogates in conservation planning^30^. However, this hypothesis is not always accepted and the role of rivers as the limits to endemism areas has been questioned for many taxa^13,17,26,27,31–41^. For example, the effects of the Tapajós River (for amphibians and squamates^17^) and the Amazon River (for birds^26^) as barriers depend on the life-history characteristics of the species. Dambrós *et al*.^13^ showed that sites separated by large geographic distances had distinct termite-species composition and most of the broad-scale variation in species composition could be explained either by spatial predictors or differences in environmental conditions between regions, and not by large rivers, such as the Madeira, Negro, Branco and Amazon.

In the majority of the studies that accepted the river-barrier hypothesis, the conclusions were based on studies with few species^20,23^ and on the assumed absence of species on one bank^16,17,24^. In addition, rivers vary in discharge and width, and these two factors have been considered important in determining when large rivers function as geographic barriers to species dispersal^24,36,42^. Therefore, the acceptance or rejection of the hypothesis may depend mainly on the species and river investigated. These two factors together make it difficult to generalize the importance of large rivers as effective geographical barriers to the distribution of Amazonian species and as a possible hypothesis to explain the species diversity found in the region.

In this study, we estimated the proportion of species in different taxonomic groups [Hymenoptera (Apidae), Hymenoptera (Formicidae), Coleoptera, Lepidoptera, Isoptera, Orthoptera, Snakes, Lizards (excluding snakes), Anura, Chiroptera, Primates, Small mammals (Didelphimorphia, Rodentia), Large mammals (Rodentia, Pilosa, Ungulados, Carnivora, Artiodactyla, Cingulata) and Birds] that have their distributions limited by a river (the generic hypothesis of the river as a barrier) and the number of species for which there is evidence (sister species on opposite banks of the river) that this river functioned as a vicariance barrier causing speciation (the hypothesis of existence of endemism areas based on large rivers). We used only species for which false absences are unlikely to explain the appearance of the river as a barrier. We conducted the study on the Madeira River, which has been postulated as a barrier to dispersal for species of various taxa^16,18,22,38,43–47^ and the border between two endemism areas^29,30^, and we studied an area in the mid reaches where many studies have indicated that it is an effective biogeographic barrier. Our results indicate that the hypothesis that the Madeira River is the border that separates two endemism areas (Inambari and Rondonia) and that the river-barrier hypothesis explains much of the diversity found in the region is inappropriate for most species, and we suggest that alternative hypotheses should be proposed to explain the limits of distributions of most species found in the region, as well as a revision of the criteria that are used to determine species-endemism areas.

## Results

### Generic hypothesis of large rivers as barriers

**Figure 1:**
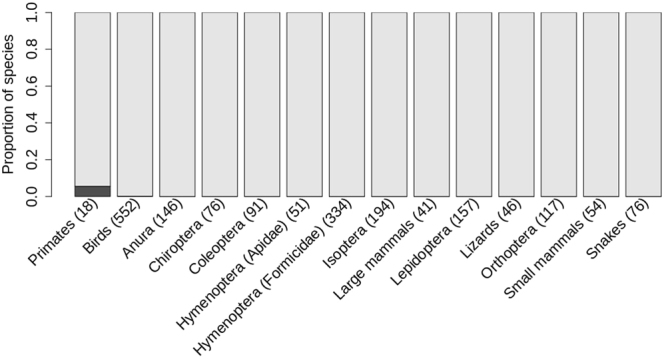
Estimates of the proportion of species with detectability >50% in each taxonomic or functional group that had their distributions limited by the Madeira River (Dark gray). Light-gray bars show the proportion of species for which the Madeira River was not a geographic barrier. Numbers in parentheses denote the number of species in each taxonomic or functional group.

The hypothesis that the distribution of species around the Madeira River is mainly related to dispersal limitation caused by river barriers, was rejected for most species studied (Fig. 1, Supplementary Table S1). Of the 1952 species with detection probabilities sufficiently high that false absences are improbable, only 0.10% (Primates: *Saguinus labiatus labiatus* and Aves:*Lepidothrix coronata*) had their distributions limited by the river (Supplementary Figs S1 and S2).

Because the proportion of species limited to one side of the river depends on our decision as to which species the detection probability was high enough for a valid test, our results might underestimate the number of species limited to one side of the river if the species that are limited by the river are those that are difficult to detect. Therefore, we report the number of species in each taxonomic or functional group that had their distributions limited by the river considering other *P*_*expected*_ in Supplementary Table S1, and give their distributions in Supplementary Figs S1 and S2 (only for species with detection probability ≥ 0.40). The number of species apparently separated by the river was low in all cases, except when we made absolutely no correction for probable false absences (Supplementary Table S1).

### Hypothesis of the existence of endemism areas based on large rivers

**Figure 2:**
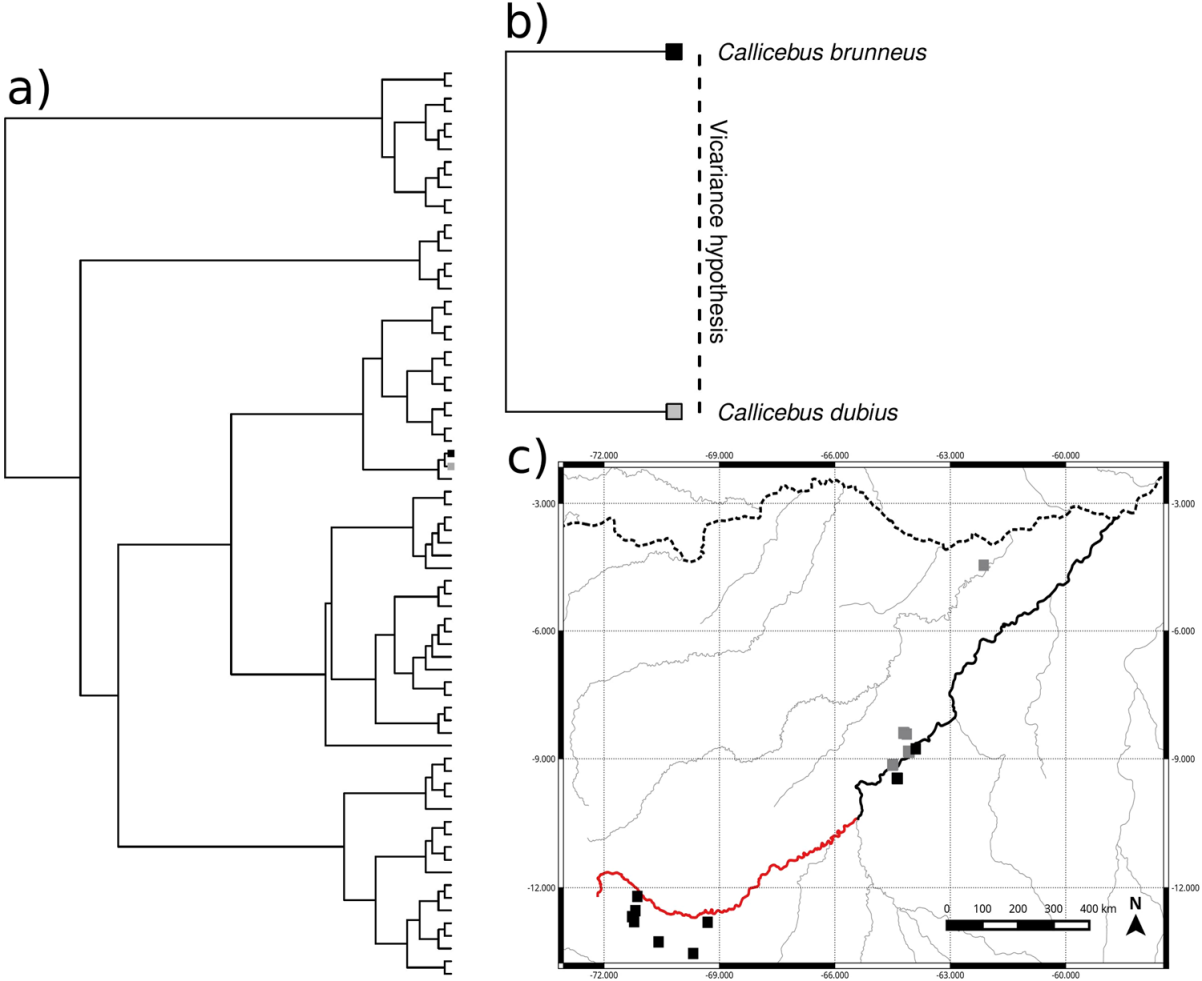
Evidence suggesting that the Madeira River could have functioned as a vicariance barrier for *Callicebus brunneus* and *Callicebus dubius*. (**a)** Phylogenetic hypothesis of small, large and non-flying mammals (72 spp); (**b)** Vicariance hypothesis; and (**c)** Species distributions along the Madeira River; black squares represent known occurrence of *C*. *brunneus*, and gray squares represent known occurrence of *C*. *dubius;* the black solid line represents the Madeira River; the red solid line represents the Madre de Dios River in Bolivia and the dashed line represents the Amazon River. See Supplementary Fig. S7 for detailed phylogenetic hypotheses associated with species distributions along Madeira River (right or left bank of the river). Map generated using QGIS v2.18 (http://www.qgis.org).

**Figure 3:**
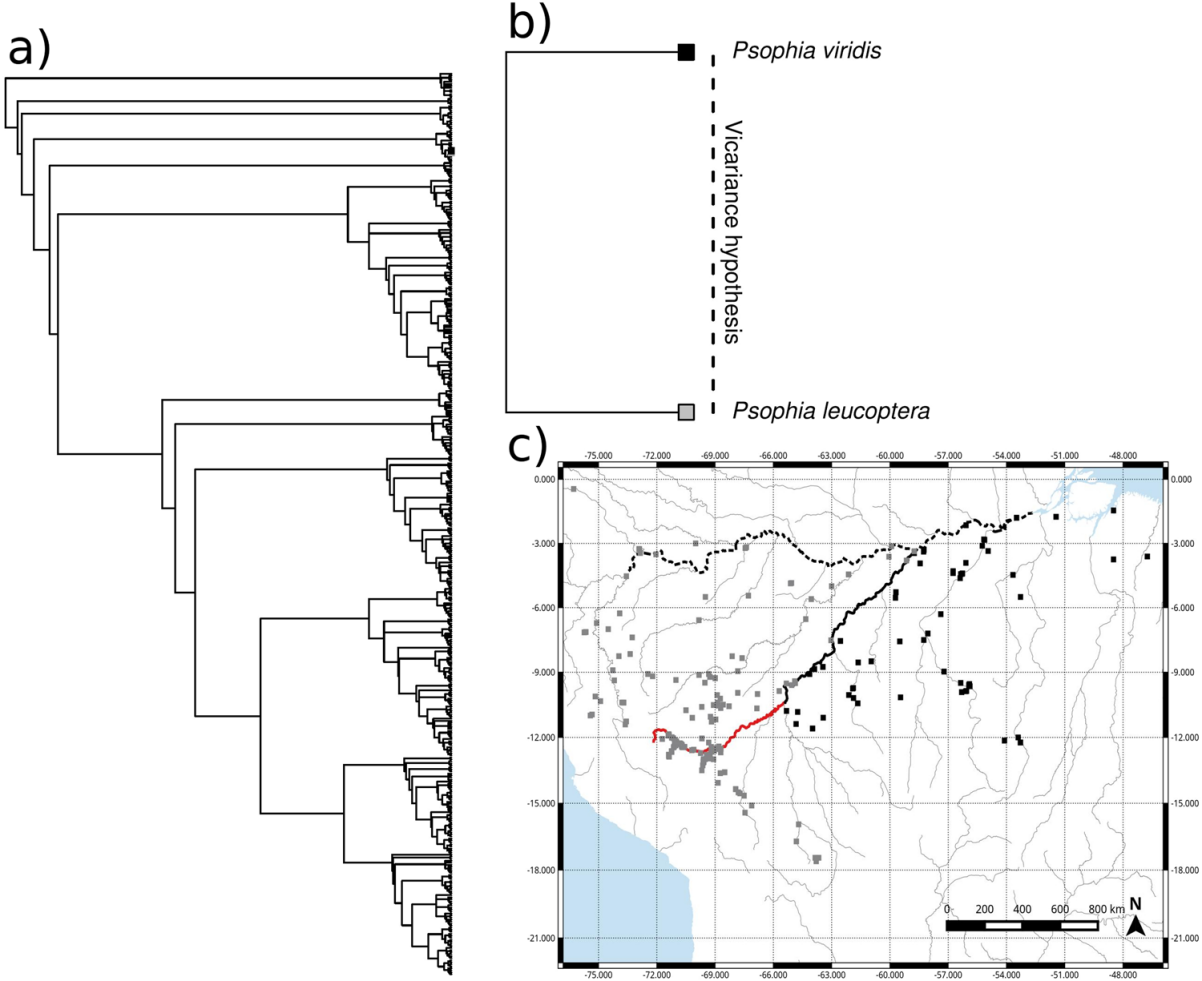
Evidence suggesting that the Madeira River could have functioned as a vicariance barrier for *Psophia viridis* and *Psophia leucoptera*. (**a)** Phylogenetic hypothesis of Aves (446 spp); (**b)** Vicariance hypothesis; and (**c)** Species distributions along the Madeira River; black squares represent known occurrence of *P*. *viridis* and gray squares represent known occurrence of *P*. *leucoptera;* the black solid line represents the Madeira River; red solid line represents the Madre de Dios River in Bolivia; and the dashed line represents the Amazon River. See Supplementary Fig. S8 for detailed phylogenetic hypotheses associated with species distributions along the Madeira River (right or left bank of the river). Map generated using QGIS v2.18 (http://www.qgis.org).

Evidence that the Madeira River works as a vicariance barrier causing speciation (presumption of the endemism-areas hypothesis) was not found for 713 (99.45%) of the species investigated for which we could obtain data to erect robust phylogenetic hypotheses (Supplementary Figs S3–S7). We found evidence suggesting that the river had functioned as a vicariance barrier only for 4 (0.55%) of the species [Primates: *Callicebus brunneus* e *Callicebus dubius* (Fig. 2) and Aves: *Psophia viridis* e *Psophia leucoptera* (Fig. 3)].

## Discussion

The hypothesis of the Madeira River as the limit of distribution was not supported for most species, so our results are not concordant with the river-barrier hypothesis explaining the origin^20,22^ or spatial-distribution limits of species^16,17^, nor of the existence of endemism areas^29,30^, for most of the species that occur around the Madeira River. Even if the hypothesis is correct that the effectiveness of the river as a barrier depends on the characteristics of life histories of the species for a small proportion of some taxa^17,26^, this would explain only a very small part of the biological diversity of the Amazon^40,48,49^.

In most studies that accepted the hypotheses about the effects of rivers^16,17,24^, the apparent absence of a species on the opposite bank to that sampled was used to conclude that a large river was a geographical barrier. However, any species-sampling technique has some bias and the absence of a species in a certain location might indicate that the species was simply not detected^50^.

For example, Dias-Terceiro *et al*.^16^ found that the distribution of *Ameerega trivittata* (Anura:Dendrobatidae) was restricted to the left bank of the Madeira River (accepting the generic hypothesis of large rivers). This species was recorded on both banks in the Madeira River in our study and also on the left bank of the Tapajós River in the study by Moraes *et al*.^17^. The Tapajós River is located adjacent to the right bank of the Madeira River, and the presence of a species on the left bank of the Tapajós River implies the presence of *A*. *trivittata* on the right bank of the Madeira River. Fecchio *et al*.^47^ concluded that the composition of parasites in birds was dependent on endemism areas in the Amazon, but some of the host species that supported this conclusion occurred in our samples independent of the endemism area. It is possible that the conclusion of these authors was biased by the false absence of the host in one of the areas of endemism. This possible bias in conclusions has been observed for other species in Cracraft^29^, a reference that has been widely used to support and justify studies that determine endemism areas in the Amazon, based only on the apparent absence of a species on the opposite bank of a large river. It is possible that these are not the only cases of doubtful results in the literature, since this type of potential error was detected many times in our analyses. In approximately 40% of the species, the detectability analysis indicated that sampling was inadequate to draw a conclusion. It could be that only hard-to-detect species are affected by rivers, but this seems unlikely since the river-barrier hypotheses were raised based on easily-detected species.

It is unquestionable that large rivers are the distribution limits of some Amazonian species, but the large number of exceptions indicates that the indication of the Madeira River as a border between endemism areas may be inappropriate for most species. It is important to emphasize that rivers can function as species limits without necessarily indicating that they represent barriers that caused vicariance speciation^51^, an assumption of the existence of endemism areas based on large rivers. Alternatively, sympatric speciation via sexual selection^52,53^, environmental differences^54–56^ or ecological interactions^57,58^; combined with dispersal limitation^51,59^ and competition^60^ could produce the same patterns of allopatric distribution observed in Figs 2 and 3, and also in Ribas *et al*.^22^, Fernandes *et al*.^20^, Boubli *et al*.^23^ and are likely more important mechanisms for generating and maintaining Amazonian biodiversity than rivers. However, these alternative hypotheses are often ignored in studies that accept the hypothesis of large rivers as the cause of speciation. Moreover, most of the conclusions relating to the river-barrier hypotheses assume that the geographical distribution of a species does not change over time, but there is evidence that many distributions in the past were different from current distributions^61–65^.

The lack of evidence found to support the river-barrier hypotheses (generic hypothesis of large rivers as barriers, and the hypothesis of centers of endemism based on large rivers) in a stretch of river commonly postulated as the border between endemism areas^16,18,29,30,38,44–47^, suggests that the hypothesis of existence of endemism areas based only on the distributions of a few species and very large rivers, should be reevaluated for the majority of species. With the reevaluation of these limits, the need for new hypotheses will arise to explain the Madeira River’s role in the origin and distribution of Amazonian biodiversity. More importantly, in the absence of information on the distributions of most species, the proposed endemism areas are being used as surrogates in conservation planning^30^. Substitutes should only be used when there is strong evidence of the relationship between the majority of targets and the proposed substitute^66^. In the case of centers of endemism, this evidence is not available for most Amazonian rivers, and specifically for the Madeira River, the evidence that it is a border between endemism areas applies to a very small proportion of biodiversity.

Our results are for only one area and there are taxonomic issues relating to species boundaries that need to be worked out for many taxa. Most of the species we studied are recognized on morphological criteria and with the application of molecular methods more species could be discovered that have the Madeira River as a limit to their distributions. Nevertheless, our results indicate that the roles of large rivers in promoting biological diversity and the use of postulated endemism areas as convenient surrogates for conservation planning in the Amazon still need to be tested for the particular taxonomic group and conservation question being addressed.

## Methods

### Study area

**Figure 4:**
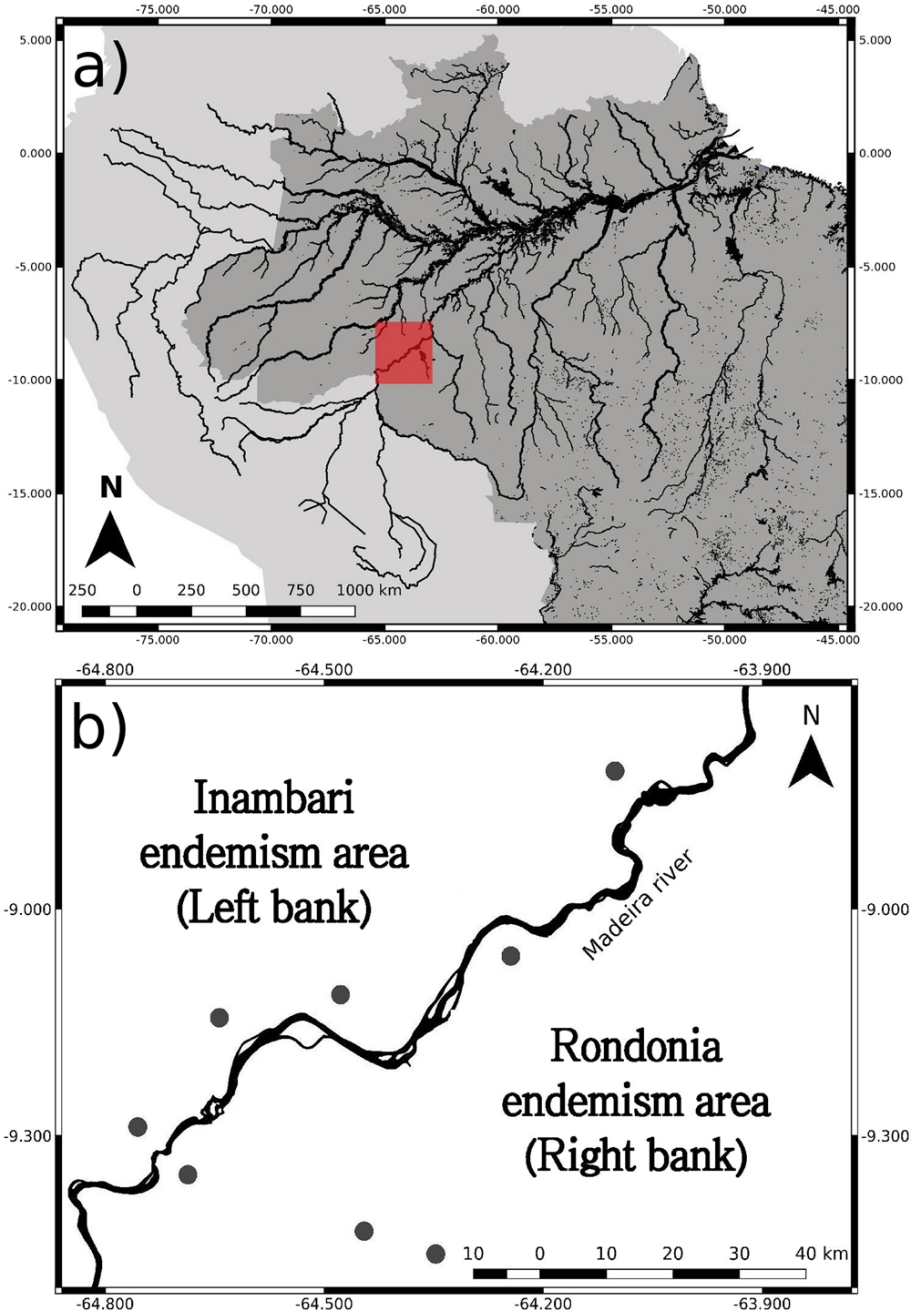
Location of study area (maps generated using QGIS v2.18, http://www.qgis.org). (**a)** Section of the river investigated (red square); and (**b)** Location of sample grids (black dots) along the Madeira River (see sample-grid details in Fig. S8).

We undertook the study along the Madeira River (Fig. 4), one of the main tributaries of the Amazon River. The section of the river investigated is in the region where the river has a width of approximately 1.6 km, which has been considered a strong barrier in many previous studies^16,18,22,38,44–47^ and the border between endemism areas^29,30^.

### Data source

To estimate the proportion of species whose distributions are effectively delimited by the river, we took advantage of an intensive study of the fauna associated with the implantation of a hydroelectric dam on the Madeira River. Sampling was carried out on both banks of the river, following the RAPELD protocol^67^ (Supplementary Fig. S8). Some species may be limited by rivers but not occur on the immediate banks due to habitat-type (e.g. flooded area) avoidance. However, the field infrastructure comprised two parallel 5-km trails (Supplementary Fig. S8) and also sampled non-flooded area. The number of samples per bank and taxonomic groups surveyed are listed in Supplementary Table S2. In this study, we investigated only the distributions of animal species, since none of the evidence used to propose the river–barrier hypothesis was based on information about plants or microorganisms.

### Data analysis

#### Generic hypothesis of large rivers as barriers

It was not possible to test the hypothesis for all the species of the region, because little is known about the distributions of many species, and many Amazonian species have not yet been described. As surveys of each taxonomic or functional group were made by the same researchers, we could include non-described species (hereafter referred to as morphospecies), for those species for which detectability analyses indicated that the absence of records on one bank of the river had little chance of being due to false absences.

In order to obtain an unbiased estimate of the proportion of species in each taxonomic or functional group [Hymenoptera (Apidae), Hymenoptera (Formicidae), Coleoptera, Lepidoptera, Isoptera, Orthoptera, Snakes, Lizards (excluding snakes), Anura, Chiroptera, Primates, Small mammals (Didelphimorphia, Rodentia), Large mammals (Rodentia, Pilosa, Ungulados, Carnivora, Artiodactyla, Cingulata) and Birds] that had their distributions limited by the river, we considered that the river was a potential geographical barrier only when detectability analyses indicated that the expected probability (*P*_*expected*_) of the species truly being absent from one of the banks (right or left) was *P*_*expected*_ ≥ 0.50. This criterion allows us to conclude that the absence of a species on the opposite bank to which it was present is unlikely to be due to false absences caused by failures in the detection of the species. This expected probability was estimated according to the formula:

$${{\rm{P}}}_{{\rm{expected}}}=1-{[1-({\rm{N}}/{{\rm{N}}}_{{\rm{sampleBank}}})/{{\rm{N}}}_{{\rm{sampleBank}}}]}^{{\rm{NsampleOppositeBank}}}$$

where: *P*_*expected*_, is the expected probability of the species occurring on the bank opposite to that on which it was recorded; *N* is the number of samples where the species occurred; *NsampleBank* is the total number of samples on the bank where the species was present (right or left bank); *NsampleOppositeBank*, is the total number of samples on the opposite bank to which the species was recorded.

#### Hypothesis of the existence of centers of endemism based on large rivers

The hypothesis of the existence of endemism areas based on large rivers was tested for 717 species (no false absences taken into account) of vertebrates for which it was possible to obtain phylogenetic information. To indicate if the river worked as a vicariance barrier independent of the taxonomic or functional group, we constructed a phylogenetic hypothesis separately for each group (Figs S3–S7). For small, large and non-flying mammals (72 spp), snakes (66 spp), lizards (35 spp) and frogs (98 spp), the phylogenetic relationships were obtained with the R package “rotl”^68^, and for birds (446 spp) the information was obtained through the website birdtree.org^69–71^.

To determine the number of sister species or lineages for which the river was an apparent vicariance barrier, we associated each species in the phylogenetic hypotheses (referring to the different taxonomic or functional groups) with their location of occurrence (right or left bank of the river). If sister species or lineages (indicated by the phylogenetic hypothesis) were present on opposite banks (allopatric distribution), this result could be an indication that the river functioned as a vicariance barrier.

#### Avoiding potential sample biases

Before accepting the generic hypothesis of large rivers as barriers, and the hypothesis of existence of endemism areas based on large rivers, and to minimize the effect of sampling on the results, we checked the distribution of each species that apparently occurred only on one bank based on the data from Santo Antônio with records in the literature and in the websites of the Global Biodiversity Information Facility (http://www.gbif.org), *speciesLink* (http://www.splink.org.br, Information system that integrates in real time, primary data of scientific collections), *Portal da Biodiversidade* (https://portaldabiodiversidade.icmbio.gov.br/portal/, this site provides data and information on Brazilian biodiversity generated or received by the Ministry of the Environment and related institutions) and the Smithsonian National Museum of Natural History (https://naturalhistory.si.edu/).

### Data Availability

The datasets analyzed during the current study were collected during the environmental-impact studies for the Santo Antônio hydro-electric reservoir and are of open-access through the web site of the Instituto Brasileiro do Meio Ambiente e dos Recursos Naturais Renováveis (IBAMA) web site. However, due to some inconsistencies in that data base, the data used here were provided by Santo Antônio Energia and were further quality checked. They are available from the corresponding author on request.

## Electronic supplementary material

Supplementary information

Supplementary Dataset
