# Supplementary material for: Most species are not limited by an Amazonian river postulated to be a border between endemism areas
Source: Sci Rep. 2018 Feb 2;8:2294. doi: 10.1038/s41598-018-20596-7 (PMC5797105; doi:10.1038/s41598-018-20596-7)

**Most species are not limited by an Amazonian river postulated to be a border between endemism areas**

Sergio Santorelli Junior^1,*^, William E. Magnusson^2,3,+^, Claudia Deus^3,+^

^1^Programa de Pós graduação em Ciências Biológicas, Instituto Nacional de Pesquisas da Amazônia, Av. André Araújo, 2.936, Petrópolis, CEP 69.067-375, Manaus, Amazonas, Brazil.

^2^Centro de Estudos Integrados da Biodiversidade Amazônica, Av. André Araújo, 2.936, Petrópolis, CEP 69.067-375, Manaus, Amazonas, Brazil.

^3^Coordenação de Pesquisas em Biodiversidade, Instituto Nacional de Pesquisas da Amazônia, Av. André Araújo, 2.936, Petrópolis, CEP 69.067-375, Manaus, Amazonas, Brazil.

*santorelli.jr@gmail.com

^+^these authors contributed equally to this work


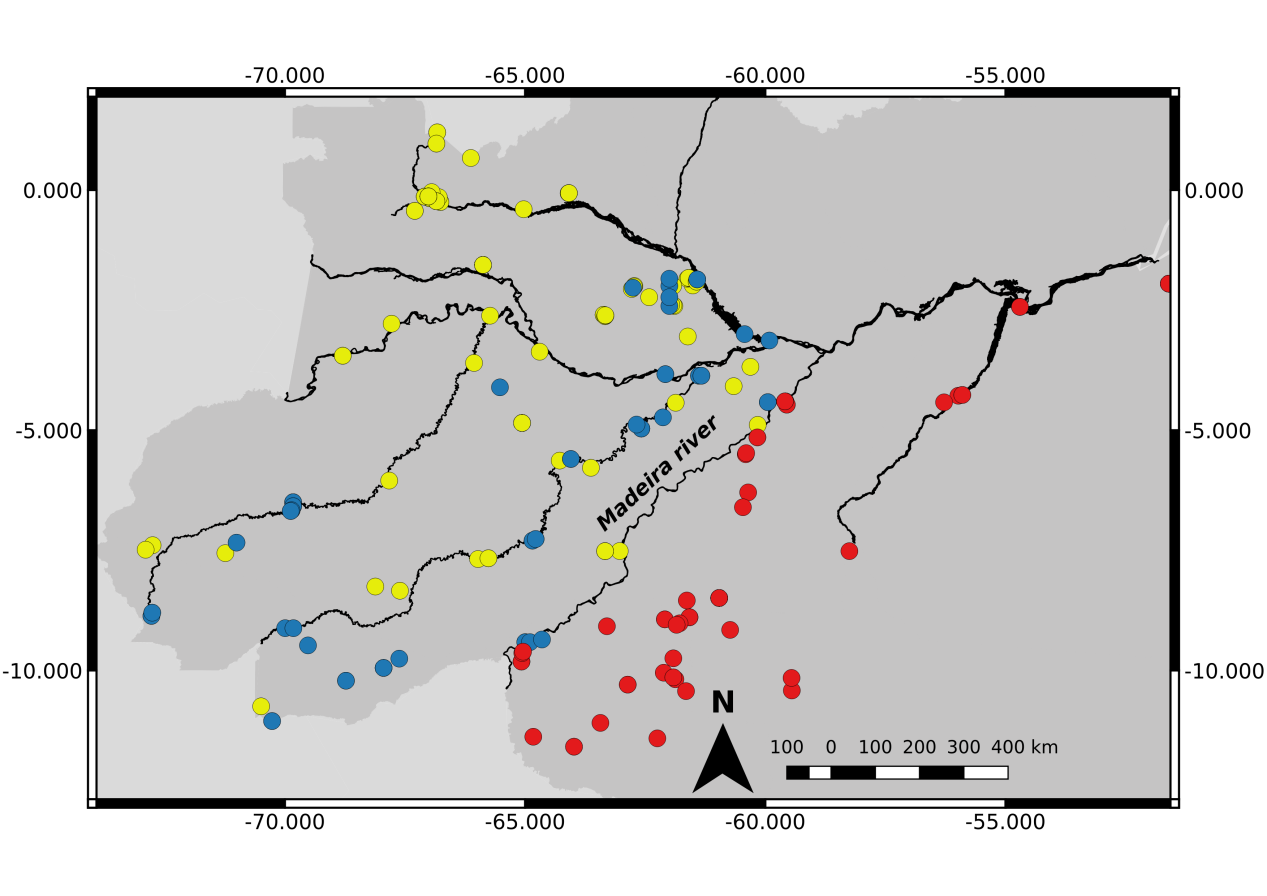


**Figure S1** - Species distributions limited by the Madeira River; yellow dots represents current distributions of *Lepidothrix coronata;* blue dots represents current distributions of *Hypocnemis peruviana*; and red dots represents current distributions of *Rhegmatorhina hoffmannsi.* Map generated using QGIS v2.18 (http://www.qgis.org)

*
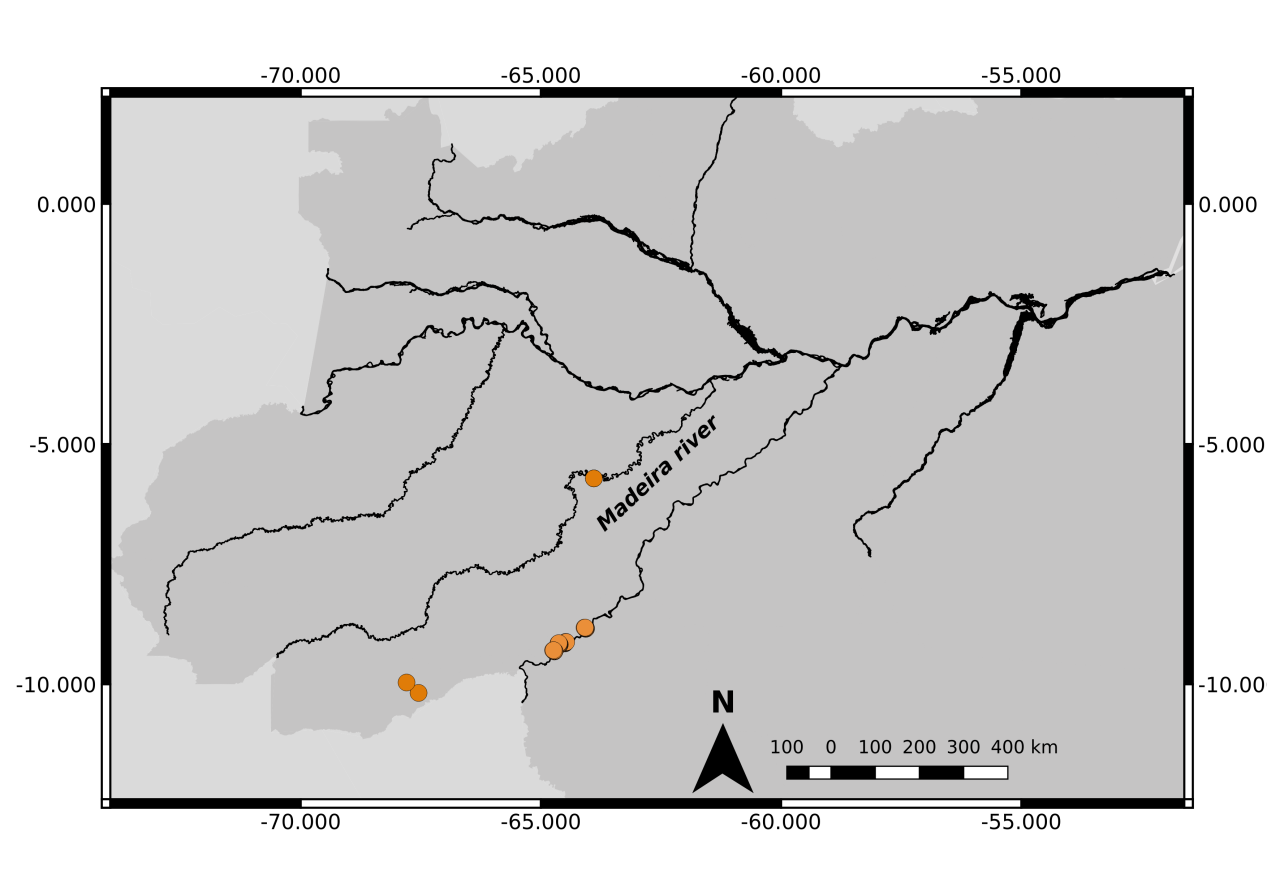
*

**Figure S2** *-* Distribution of *Saguinus labiatus labiatus* (Orange dots) limited by the Madeira River. Map generated using QGIS v2.18 (http://www.qgis.org)


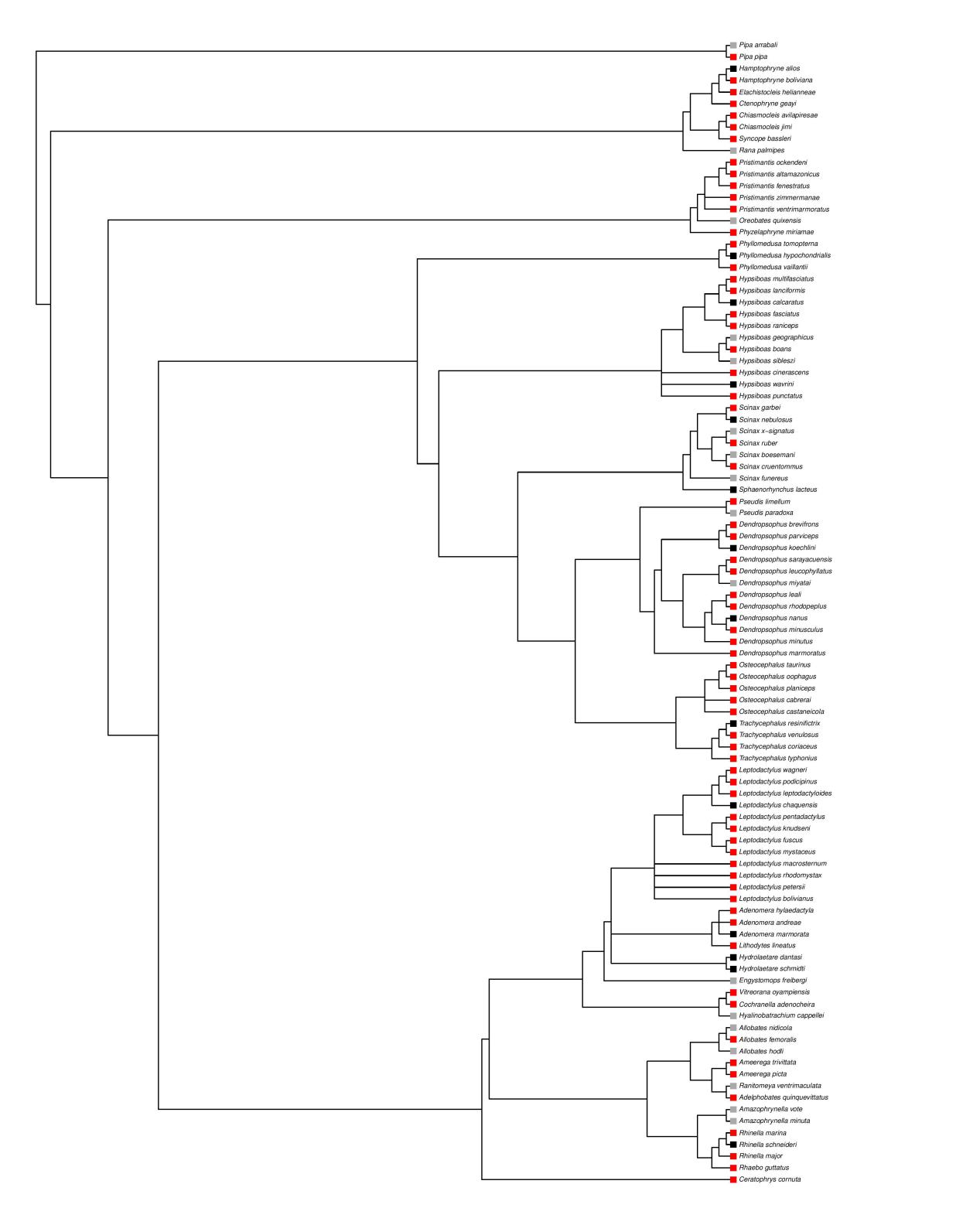


**Figure S3** - Phylogenetic hypothesis for anuran species (98) with distributions limited by or crossing the Madeira River; red squares indicate species recorded on both banks of the river; black squares indicate species recorded only on the right bank of the river (Rondonia endemism area); and gray squares indicate species recorded only on the left bank of the river (Inambari endemism area).


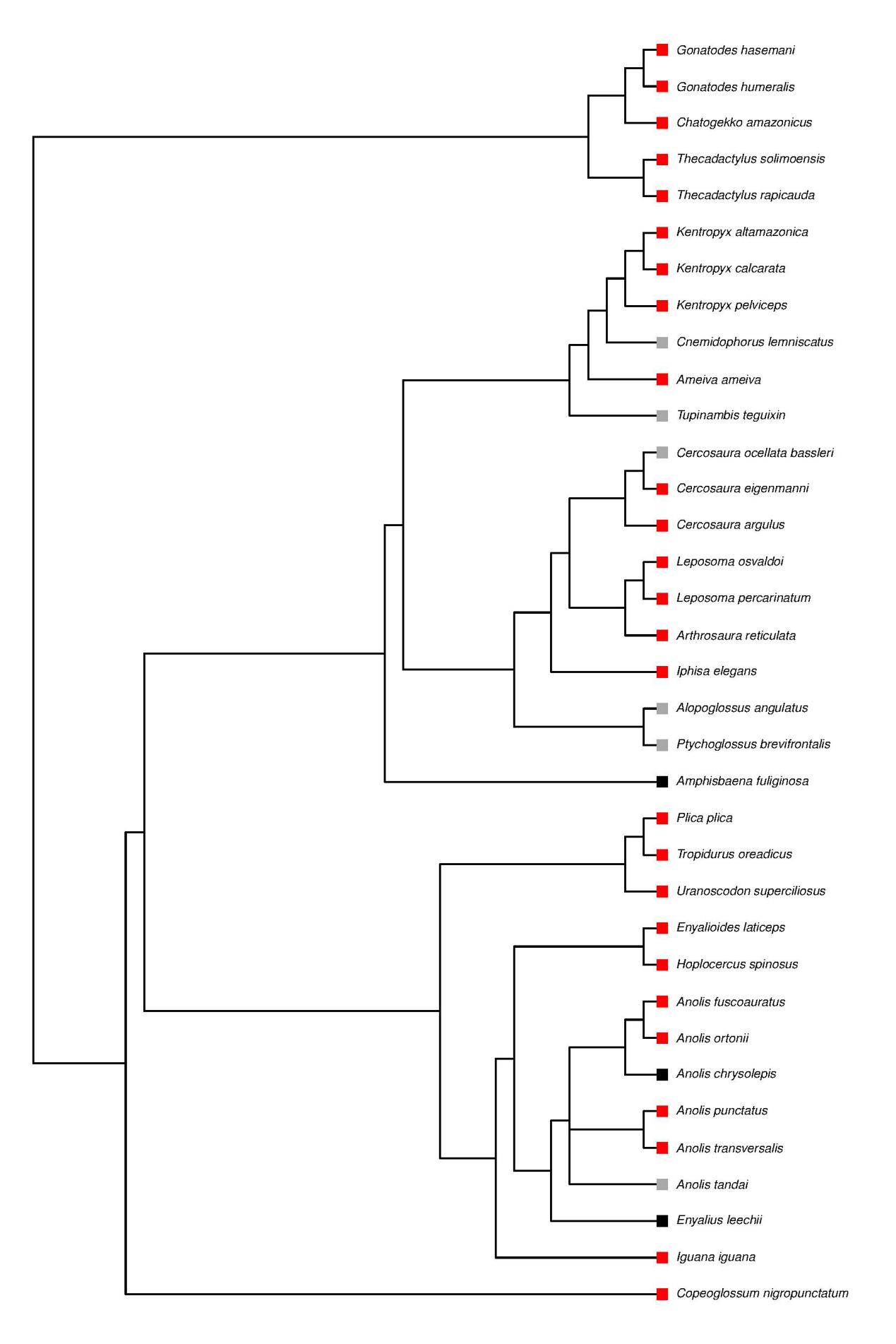


**Figure S4** - Phylogenetic hypothesis for lizards (excluding snakes) species (35) with distributions limited by or crossing the Madeira River; red squares indicate species recorded on both banks of the river; black squares indicate species recorded only on the right bank of the river (Rondonia endemism area); and gray squares indicate species recorded only on the left bank of the river (Inambari endemism area).


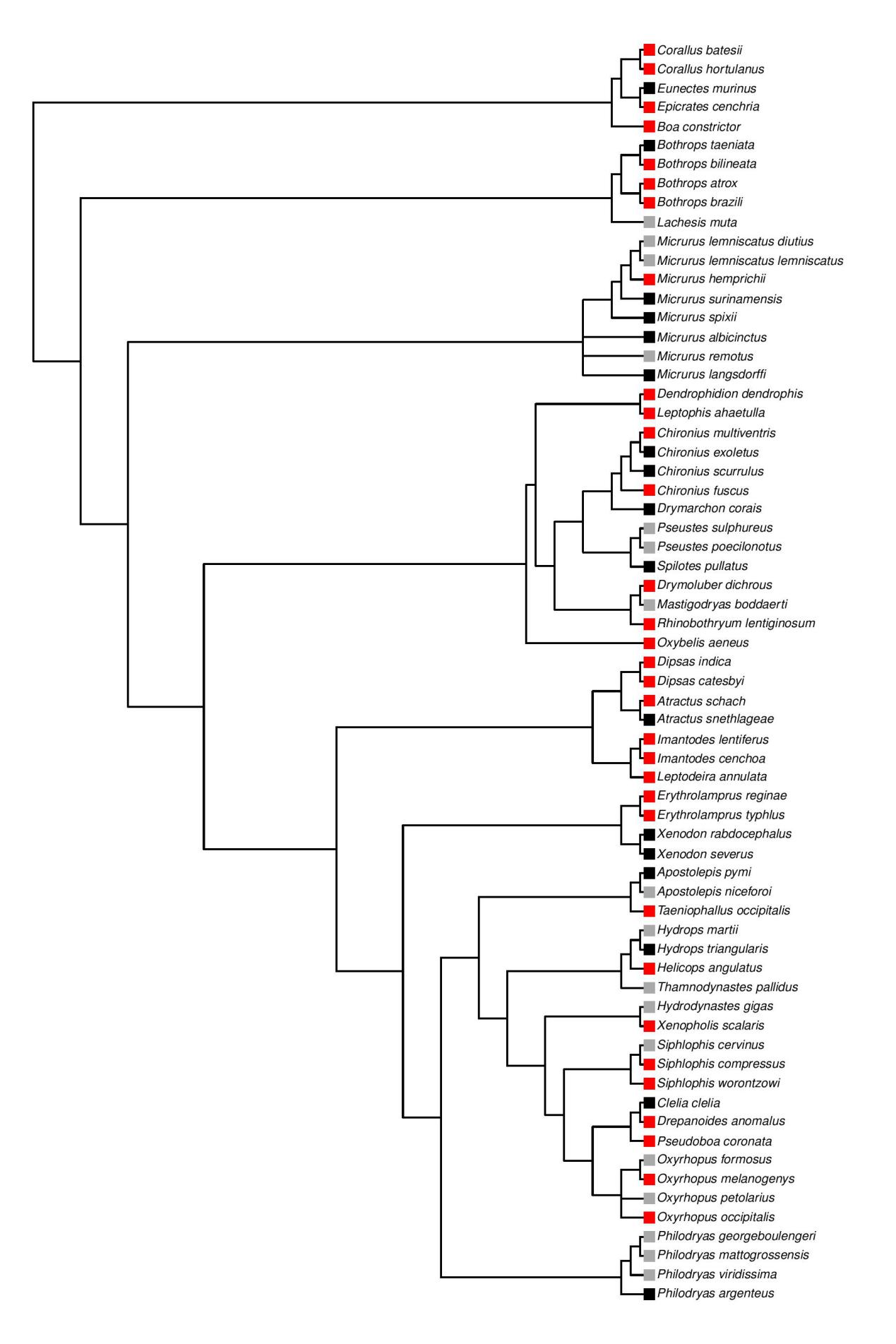


**Figure S5** - Phylogenetic hypothesis for snakes species (66) with distributions limited by or crossing the Madeira River; red squares indicate species recorded on both banks of the river; black squares indicate species recorded only on the right bank of the river (Rondonia endemism area); and gray squares indicate species recorded only on the left bank of the river (Inambari endemism area).
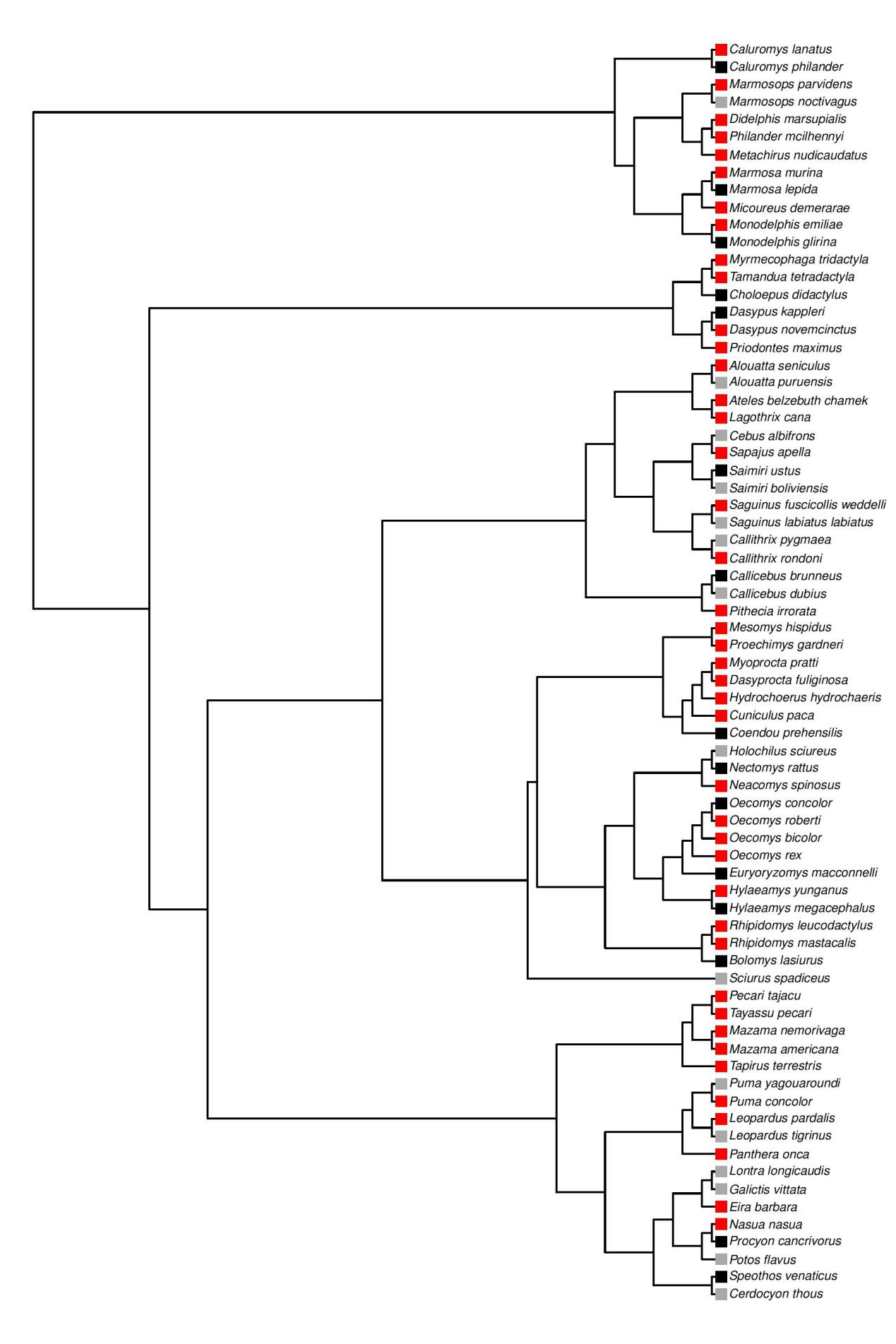


**Figure S6** - Phylogenetic hypothesis for small and large non-flying mammals species (72) with distributions limited by or crossing the Madeira River; red squares indicate species recorded on both banks of the river; black squares indicate species recorded only on the right bank of the river (Rondonia endemism area); and gray squares indicate species recorded only on the left bank of the river (Inambari endemism area).


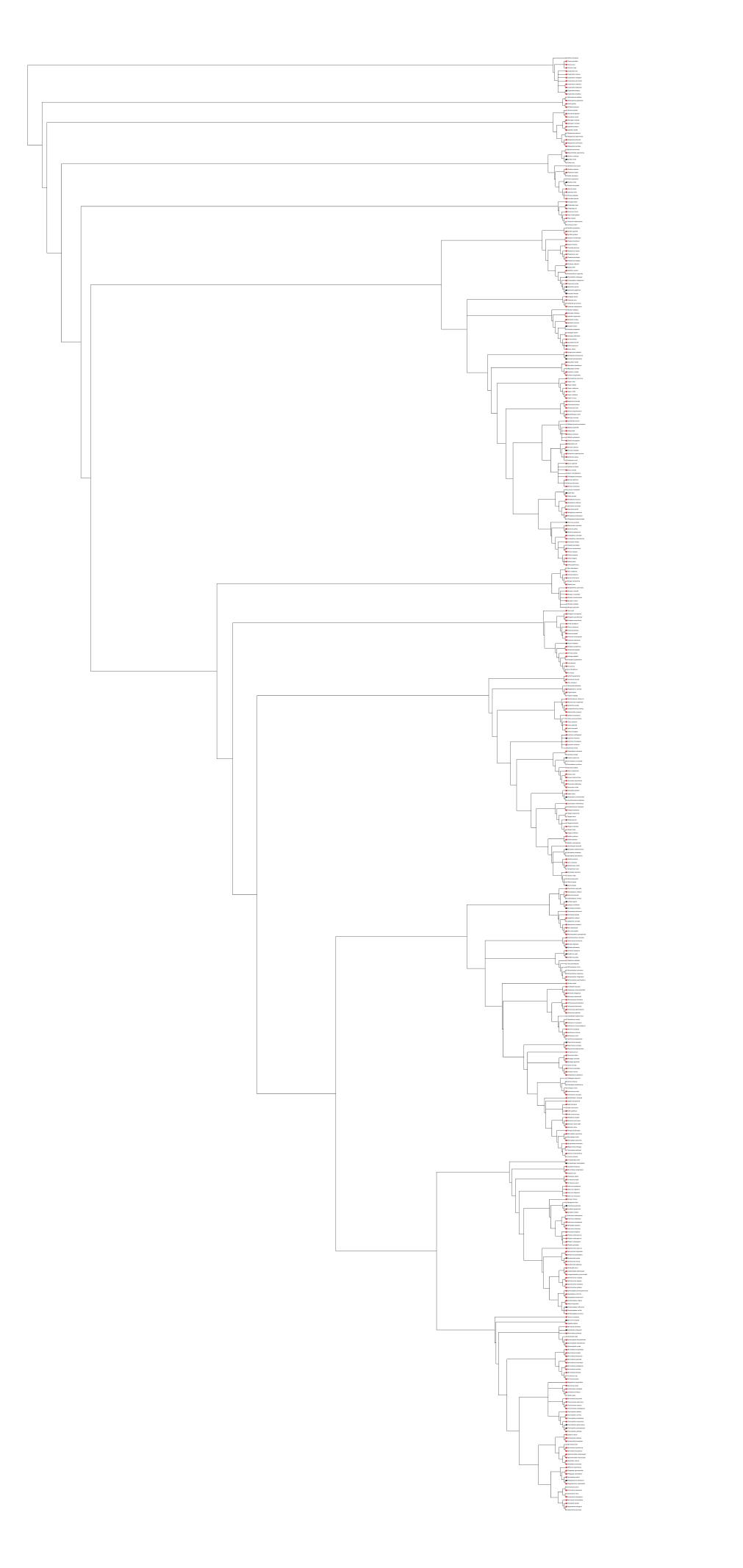


**Figure S7** - Phylogenetic hypothesis for Aves species (446 spp) with distributions limited by or crossing the Madeira River; red squares indicate species recorded on both banks of the river; black squares indicate species recorded only on the right bank of the river (Rondonia endemism area); and gray squares indicate species recorded only on the left bank of the river (Inambari endemism area).


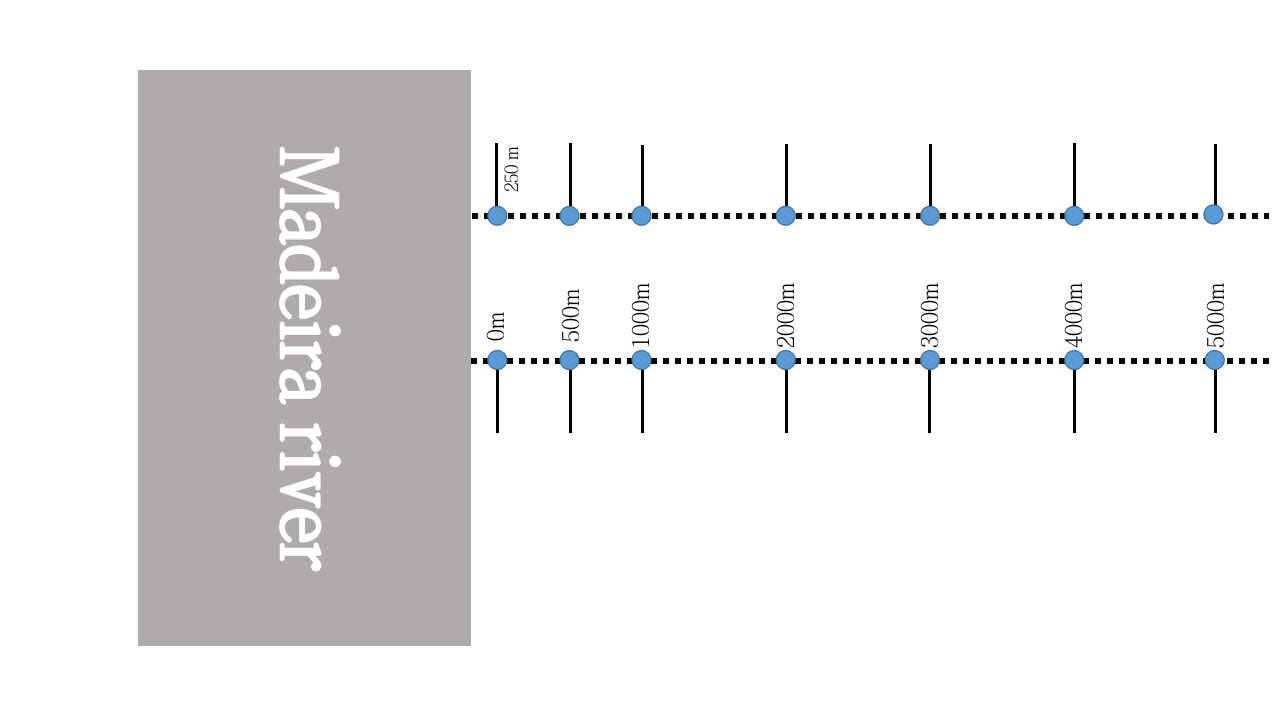


**Figure S8** - Sample grid details. Each grid (black dots in Fig.1B) was composed of two parallel 5-km long trails (dashed line) with 14 permanent sampling plots (blue dots) positioned 0, 500, 1000, 2000, 3000, 4000 and 5000 m from the river bank. Blue line indicate the limit of the flooded area in Madeira River.


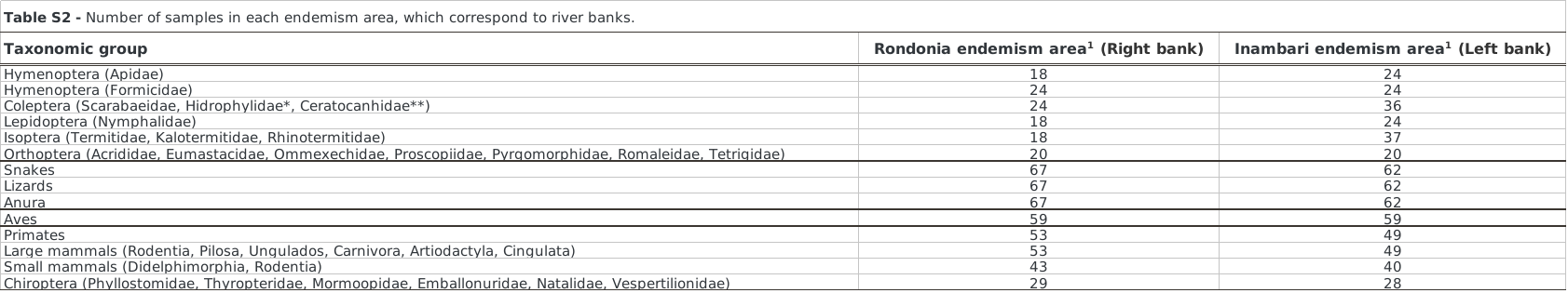

Supplement: Supplementary file 1 — Supplementary information [file 41598_2018_20596_MOESM1_ESM.doc]
